# Boosting the Separation of Adeno-Associated Virus Capsid Proteins by Liquid Chromatography and Capillary Electrophoresis Approaches

**DOI:** 10.3390/ijms24108503

**Published:** 2023-05-09

**Authors:** Megane K. Aebischer, Thomas Bouvarel, Emmalyn Barrozo, Dominik Kochardt, Carsten Elger, Markus Haindl, Raphael Ruppert, Davy Guillarme, Valentina D’Atri

**Affiliations:** 1 School of Pharmaceutical Sciences, University of Geneva, CMU-Rue Michel Servet 1, 1211 Geneva, Switzerland; 2 Institute of Pharmaceutical Sciences of Western Switzerland, University of Geneva, CMU-Rue Michel Servet 1, 1211 Geneva, Switzerland; 3 Roche Diagnostics GmbH, Nonnenwald 2, 82377 Penzberg, Germany

**Keywords:** adeno-associated virus, liquid chromatography, step gradient, multi-isocratic conditions, gene therapy, viral particles

## Abstract

The purity of the three capsid proteins that make up recombinant adeno-associated virus (rAAV) is considered a critical quality attribute of gene therapy products. As such, there is a clear need to develop separation methods capable of rapidly characterizing these three viral proteins (VPs). In this study, the potential benefits and limitations of different electrophoretic and chromatographic methods were evaluated, including capillary electrophoresis–sodium dodecyl sulfate (CE-SDS), reversed phase liquid chromatography (RPLC), hydrophilic interaction chromatography (HILIC), and hydrophobic interaction chromatography (HIC), for the analysis of VPs obtained from different serotypes (i.e., AAV2, AAV5, AAV8, and AAV9). CE-SDS is considered to be the reference method and provides a suitable separation of VP1-3 proteins using generic conditions and laser induced fluorescence detection. However, the characterization of post-translational modifications (i.e., phosphorylation, oxidation) remains difficult, and species identification is almost impossible due to the lack of compatibility between CE-SDS and mass spectrometry (MS). In contrast, RPLC and HILIC were found to be less generic than CE-SDS and require tedious optimization of the gradient conditions for each AAV serotype. However, these two chromatographic approaches are inherently compatible with MS, and were shown to be particularly sensitive in detecting capsid protein variants resulting from different post-translational modifications. Finally, despite being non-denaturing, HIC offers disappointing performance for viral capsid proteins characterization.

## 1. Introduction

Conventional therapies are sometimes unsuccessful in treating certain genetic diseases. Therefore, intervening at the genetic level can be a valuable strategy. For this reason, gene therapy has recently changed the way genetic diseases are treated [1].

The FDA defines gene therapy as any therapy involving the modification or manipulation of the gene expression, or the alteration of the biological properties of living cells for therapeutic use. There are several possibilities to act at the genetic level [2], but one of the most common is to use vectors to introduce a gene (or transgene) directly into human cells, so that the cell can translate the gene into a functional protein. One of the most promising vectors is a viral vector known as an adeno-associated virus (AAV) [3]. AAV is made of a shell of protein called capsid encompassing a single stranded DNA of about 4.8 kb in size. Luxturna [4], Zolgensma [5], and Hemgenix [6] (for the treatment of retinal dystrophy, spinal muscular atrophy, and hemophilia B, respectively) are AAV-based gene therapies that have received FDA approval in recent years. Today, more than 300 clinical trials of AAV products are ongoing [7].

These viral vectors have many advantages. Their simple structure facilitates the production of a recombinant AAV (rAAV), in which the viral DNA is replaced by the transgene [8]. AAVs are also less immunogenic than other viral vectors [9], and they allow long-term gene expression [10]. In addition, each of the twelve serotypes has a specific tropism for certain tissues [11,12,13]. However, one of the major disadvantages of using AAVs in gene therapy is the pre-existing immunity to the viral capsids, but solutions have recently been found to overcome this immune barrier [14,15].

Because a specific AAV serotype will transduce a specific cell type or organ, the AAV serotype has a direct impact on the efficacy and safety of clinical rAAV gene therapies. Therefore, rAAV serotype identity testing is required [16]. One of the critical quality attributes (CQAs) is the characterization of rAAV viral particles (VPs) from the capsid (i.e., VP1, VP2, and VP3), which is directly related to the serotype identity. The most common analytical techniques used to investigate the serotype identity are enzyme-linked immunosorbent assay (ELISA) [17] or sodium dodecyl sulfate-polyacrylamide gel electrophoresis (SDS-PAGE) [18]. More recently, capillary electrophoresis-sodium dodecyl sulfate (CE-SDS) has become the method of choice for analyzing AAV capsid proteins [19,20].

In the present work, the reference method (CE-SDS) was compared with different liquid chromatography (LC) methods, including reversed phase liquid chromatography (RPLC), hydrophilic interaction chromatography (HILIC), and hydrophobic interaction chromatography (HIC). All the CE and LC methods were fully optimized for different AAV serotypes in order to assess whether the chromatography methods could be used as orthogonal methods to CE for the characterization of AAV VPs.

## 2. Results and Discussion

### 2.1. CE-SDS

In the first instance, a reference electrophoretic strategy was used for the analysis of AAV capsid proteins (CE-SDS). One of the main limitations in the analysis of AAV products is their low protein content, which is typically in the range of 1 × 10^11^ to 1 × 10^13^ vector genomes per mL (vg/mL), corresponding to the ng/µL level, which is about 4 to 5 orders of magnitude lower than the typical concentration of a monoclonal antibody (mAb) [21,22]. Consequently, the UV-based detection method is not sensitive enough to detect AAV capsid proteins in combination with capillary electrophoresis. Therefore, the capsid proteins were first labelled with a commercial dye (FQ-Dye) (simple and fast sample preparation procedure) and then detected by laser induced fluorescence (LIF) using a 488 nm solid-state laser and a 600 nm emission filter. This procedure enables a reduction of background noise combined with an excellent signal intensity of the protein-dye complex, allowing the monitoring of low-level impurities [23].

This strategy was applied for the analysis of viral proteins (VPs) obtained from four different serotypes, namely AAV2, AAV5, AAV8, and AAV9. It is important to note that each AAV product is composed of three VP of different sizes. Indeed, VP1, VP2, and VP3 are approximately 87 kDa, 72 kDa, and 62 kDa, respectively. Based on these differences, the separation of VP using a size-based electrophoretic separation mode (CE-SDS) should be straightforward, with VP3 eluting first, followed by VP2 and VP1. In addition, the ratio of VP subunits in the AAV capsid is known to be 1:1:10 for VP1:VP2:VP3, so the peak area corresponding to VP3 is expected to be about 10-fold larger than that corresponding to VP1 and VP2. Identification of the peaks in CE-SDS can be based on migration time (depending on VP size) and peak intensity (depending on ratio of VP subunits in the AAV product).

**Figure 1:**
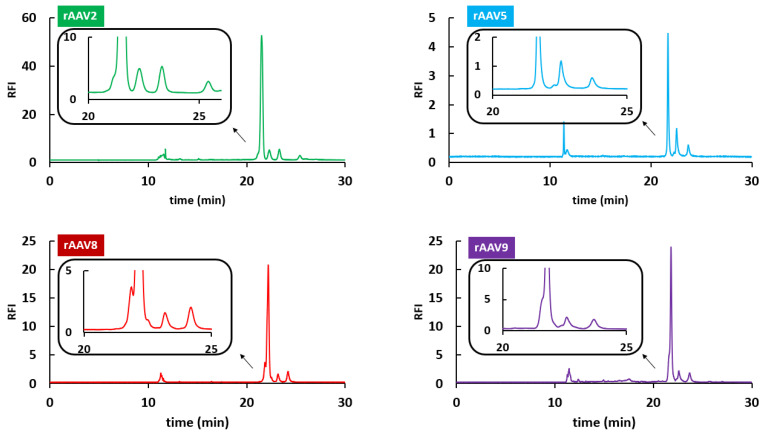
Characterization of rAAV VPs from rAAV2 (green), rAAV5 (blue), rAAV8 (red), and rAAV9 (purple) samples by CE-SDS-LIF.

As shown in Figure 1, all AAV serotypes showed different peaks, corresponding to either VP1-3 peaks, the truncated versions of the VP1-3 subunits, or other impurities present in the sample. The maximum analysis time was fixed at 30 min. For the AAV2 serotype, the three VP species migrated between 21 and 24 min (migration order: VP3–VP2–VP1). An additional species was partially separated in the front of the large VP3 peak but with an insufficient resolution. Some additional protein species were also observed between 25 and 28 min, possibly corresponding to truncated species, due to their later migration time (lower molecular weight species). For the AAV5 serotype, the three main peaks corresponding to VP1, VP2, and VP3 migrated again between 21 and 24 min. As the size of the VP subunits was similar between all AAV products, the migration window of the VPs remains comparable for all serotypes. For AAV5, a minor impurity was observed in front of the VP2 peak but again the resolution was found to be insufficient. For AAV8, the three VP peaks were well separated, and no truncated species were observed (no peak eluted after 25 min). Only two additional impurities were observed on the AAV8 product at the front and tail of the VP3 peak. Again, the resolution was found to be insufficient, and these impurities were not baseline resolved. Finally, the same electrophoretic profile was observed for the AAV9 sample, with three main peaks corresponding to VP3, VP2, and VP1, and having migration times comprised between 21 and 24 min. Only one additional impurity was observed in this sample (in front of the VP3 peak).

It is important to mention that this CE-SDS method can be considered highly generic (applicable to any AAV sample) and compatible with a regulated quality control (QC) environment. However, it remains difficult, if not impossible, to identify the minor impurities contained in the product due to the lack of MS compatibility, although a solution has recently been proposed [24,25]. For this reason, LC can be considered as an attractive alternative to CE-SDS.

### 2.2. RPLC

An RPLC-FLD method was developed with the aim of achieving the chromatographic resolution of the three main viral proteins (VP1, VP2, and VP3). First, the generic RPLC gradient conditions reported in Section “*RPLC Conditions*” were used to screen four different stationary phases (Table S1). Among the tested columns, there was a polyphenyl material (BioResolve RP mAb), two C4 columns from different providers (ACQUITY BEH C4 and YMC-Triart Bio C4), and a monolithic column based on organic polymer (ProSwift RP-4H). These wide pore stationary phases have already successfully been used for the analysis of large therapeutic proteins (i.e., mAbs or related subunits having size of 25–150 kDa). Since VPs are proteins of around 60–90 kDa, the same columns could possibly be employed. Besides their functionalization, all the tested stationary phases differed in terms of column dimensions (length of 50–150 mm and internal diameter of 1.0–2.1 mm I.D.), particle sizes (1.7–2.7 μm), and/or pore sizes (300–450 Å).

**Figure 2:**
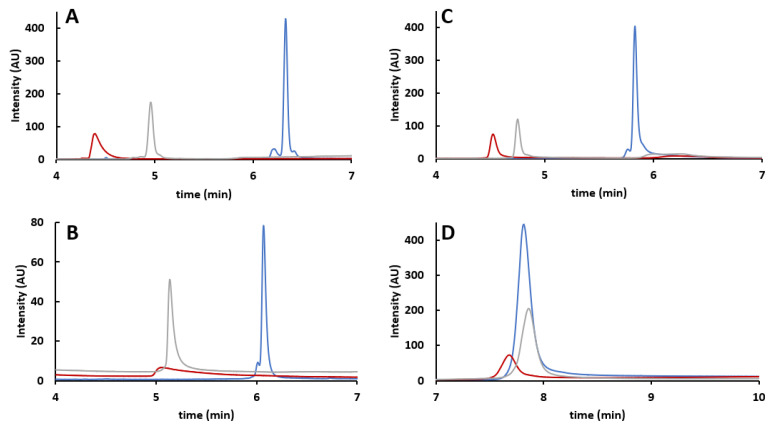
Effect of different columns and additives on RPLC analysis of rAAV8 VPs: comparison of RPLC column performance and of different additives on separation efficiency with a generic 10 min linear gradient from 20 to 60%B in 10 min on (**A**) Acquity UPLC Protein BEH C4 column, (**B**) BioResolve RP mAb Polyphenyl column, (**C**) YMC-Triart Bio C4 column, (**D**) ProSwift RP-4H column. Color code refers to the mobile phase additive, namely 0.1% TFA (blue), 0.1% FA (red), and 1.0% FA (grey).

As reported in Figure 2, preliminary method optimization to select appropriate column and mobile phase additives was performed using viral proteins (VPs) obtained from rAAV8 and mobile phases consisting of either 0.1% TFA, 0.1% FA, or 1% FA in H_2_O (mobile phase A, MPA) or ACN (mobile phase B, MPB). In all cases, analyses performed with 0.1% TFA as mobile phase additive (Figure 2, blue trace) gave the best results in terms of peak shapes. Remarkable tailing and broadening were observed when using FA, even though more acceptable results were obtained when using 1% FA (Figure 2, grey line) instead of 0.1% FA (Figure 2, red line). By considering the results obtained with 0.1% TFA, the ACQUITY BEH C4 column provides the best VPs resolution and was therefore selected for the rest of the study in RPLC mode. To further optimize the separation of the VPs, three organic solvents were screened as MPB components, namely ACN, ethanol (EtOH), and isopropanol (IPA). It should be noted that there is a remarkable difference in the eluotropic strength of each MPB component. Therefore, significant retention shifts were expected, and a dedicated gradient optimization was required. Figure S1 shows the corresponding chromatograms obtained after linear gradient optimization to achieve the best possible performance, with either can (gradient from 36 to 39%B), EtOH (38 to 41%B), or IPA (27 to 30%B). The separation of the three main VPs was always obtained. However, additional VP3 variants were resolved when using ACN as MPB component. This solvent was therefore selected for the analysis of VPs derived from different rAAV serotypes (rAAV2, rAAV5, rAAV8, and rAAV9).

To boost the VPs separation once that stationary phase chemistry and mobile phase type were defined, multi-isocratic elution methods were developed by following the workflow proposed by Murisier et al. [26]. Briefly, this workflow includes three main steps: (i) optimization of the linear gradient, (ii) application of the linear gradient through the use of pre-mixed mobile phases, and (iii) application of a multi-isocratic method designed based on the elution composition of peaks defined during step (ii).

**Figure 3:**
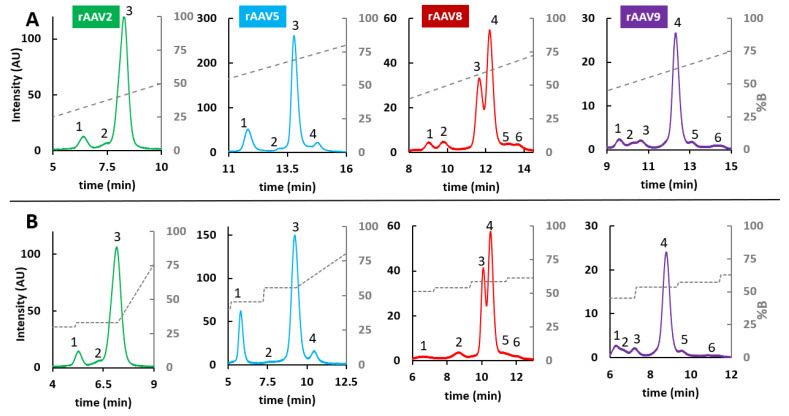
Effect of gradient type on RPLC analysis of AAV VPs: comparison of (**A**) a 20 min linear gradient 0–100%B and (**B**) a multi-step optimized gradient on separation efficiency on an Acquity UPLC Protein BEH C4 column (flow rate 0.4 mL/min, 2 µL injected, 70 °C). MPA: H_2_O/ACN (66/34 for AAV8 and 65/35 for AAV2, AAV5, and AAV9) + 0.1% TFA; MPB: H_2_O/ACN (64/36 for AAV8 and 63/37 for AAV2, AAV5, and AAV9) + 0.1% TFA. Peak numbering has the aim of highlighting the different peaks that are separated when using a linear gradient versus a multi-step gradient.

The optimization of the linear gradient performed at step (i) allowed definition of the retention window and conversion of the VPs retention times into elution compositions by using the system dwell time (t_D_) and the column dead time (t_0_). Once elution compositions were defined, they were used as initial and final compositions to run step (ii) with the optimized linear gradient using pre-mixed mobile phases. As elution compositions were generally very close, the goal of using pre-mixed mobile phases was to obtain the most accurate mobile phase delivery from the pumping system. After specific gradient optimization, VPs from rAVV8 required a MPA consisting of 0.1% TFA in H_2_O/ACN (66/34, *v*/*v*), and a MPB consisting of 0.1% TFA in H_2_O/ACN (64/36, *v*/*v*). On the other hand, VPs derived from rAVV2, rAVV5, and rAVV9 required a MPA consisting of 0.1% TFA in H_2_O/ACN (65/35, *v*/*v*), and a MPB consisting of 0.1% TFA in H_2_O/ACN (63/37, *v*/*v*). These pre-mixed mobile phases were used to run an optimized linear gradient using the elution compositions calculated as the start and end compositions of the peaks, while keeping the gradient time constant (step (ii)). Finally, for all retention times found with the optimized linear gradient, the corresponding compositions were calculated to obtain the compositions used for the isocratic steps in the multi-isocratic gradients (step (iii)). The corresponding chromatograms of both steps (ii) and (iii) are shown in Figure 3 and detailed multi-isocratic gradients are reported in Table S2.

As already reported for VPs analysis, the elution order under RPLC conditions is expected to be VP1, VP2, and VP3. In addition, the main peak is generally identified as VP3 (in line with the VPs ratio of 1:1:10 for VP1:VP2:VP3), while minor peaks eluting after VP3 are commonly assigned as fragments of VP3 protein or variants containing post-translational modifications (PTMs) such as acetylation [27]. As reported in Figure 3, a better resolution of VP1 and VP2 (assigned as peaks 1 and 2) was obtained when applying the multi-isocratic method. This enhanced separation is especially evident for VPs from rAAV8, where partially coeluted peaks 1 and 2 are baseline separated during the isocratic steps, allowing a more confident integration and eventual quantification of the peaks.

### 2.3. HILIC

An HILIC-FLD method was additionally developed with the purpose of comparing overall performance and orthogonality with the RPLC-FLD method. It should be noted that the chromatographic resolution of the three main VPs and their related variants in HILIC mode was reported on one occasion by Liu et al. [20], demonstrating the potential of this mode to chromatographically resolve VPs containing PTMs, including phosphorylation and oxidation.

**Figure 4:**
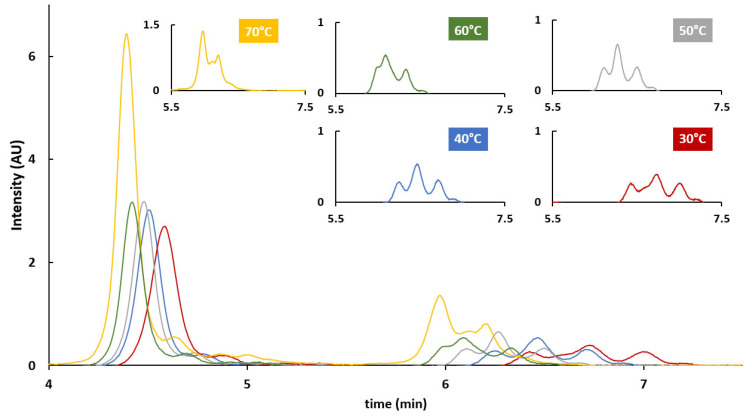
Effect of column temperature on HILIC analysis of rAAV8 VPs: comparison of 70 °C (yellow), 60 °C (green), 50 °C (grey), 40 °C (blue), and 30 °C (red) as column temperatures on a Waters ACQUITY Glycoprotein BEH Amide. MPA: H_2_O + 0.1% TFA; MPB: ACN + 0.1% TFA.

First, the generic HILIC gradient conditions reported in Section “*HILIC Conditions*” were used to assess the impact of column temperature. Column temperature is indeed a crucial parameter to ensure proper protein recovery and acceptable peak shape [28]. Generally, 70–80 °C are recommended for RPLC analysis of proteins, but it is reported that milder temperatures can be applied in HILIC mode for resolving intact proteins or related subunits [29,30,31]. VPs obtained from rAAV8 and mobile phases consisting of 0.1% TFA in H_2_O (MPA) or ACN (MPB) were used in combination with column temperatures ranging from 30 to 70 °C (considering 10 °C intervals). The corresponding chromatograms are reported in Figure 4. In accordance with the peak assignment using MS detection, reported in the literature, two sets of peaks were separated in HILIC. The first one (elution window between 4 and 5 min) corresponds to VP3 (and its possible clipped/modified variants) and the second one (elution window between 5.5 and 7.5 min) corresponds to VP1 and VP2 and their respective variants. Interestingly, the temperature decrease had just a moderate effect on peak shape and protein recovery, becoming detrimental only at 30 °C. In addition, a better resolution of the second set of peaks was obtained at 40 °C and this temperature condition was therefore selected for the rest of the study in HILIC mode.

**Figure 5:**
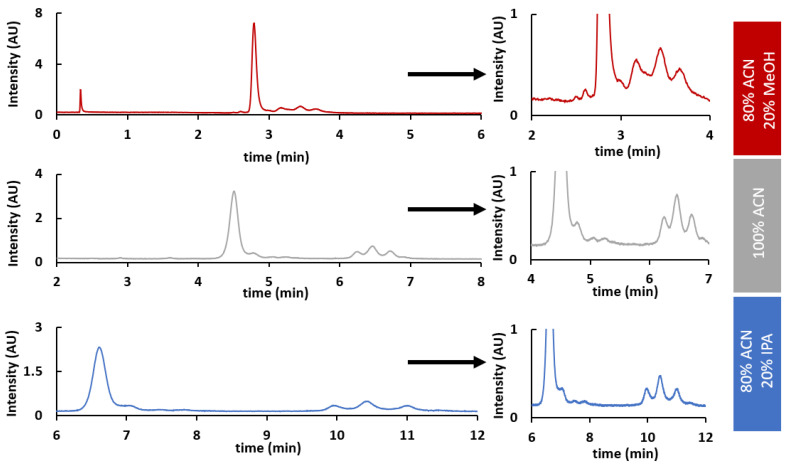
Effect of solvent selection on HILIC analysis of rAAV8 VPs: comparison of ACN, ACN/IPA, and ACN/MeOH mobile phases on a Waters ACQUITY Glycoprotein BEH Amide column with a 15 min gradient from 70 to 60%B, 75 to 60%B, and 75 to 73%B, respectively. MPA: H_2_O + 0.1% TFA; MPB: ACN + 0.1% TFA (grey), ACN/IPA (80/20, *v*/*v*) + 0.1% TFA (blue), or ACN/MeOH (80/20, *v*/*v*) + 0.1% TFA (red).

Once column temperature was defined, three different MPB compositions were screened in order to investigate their impact on selectivity. Tested conditions included an MPB consisting of 0.1% TFA in ACN, ACN/IPA (80/20, *v*/*v*), or ACN/MeOH (80/20, *v/v*). In each case, 15 min gradients were used, taking care to adapt the gradient conditions to the nature of the solvent employed, in order to obtain the best VPs separation possible. Therefore, gradients ranged from 70 to 60%, 75 to 60%, and 75 to 73% for an MPB consisting of 0.1% TFA in ACN, ACN/IPA, and ACN/MeOH, respectively. As shown in Figure 5, a better selectivity was obtained when using an MPB consisting of 0.1% TFA in ACN/IPA (80/20, *v*/*v*). Indeed, this mobile phase composition allowed a remarkable improvement in the separation of the set of peaks related to VP3 and those related to VP1 and VP2, which could be exploited to obtain a more accurate quantification.

The final step of this preliminary method optimization in HILIC mode concerned the injection volume. Injection volume is indeed one of the most crucial parameters to ensure proper protein loading on the column and acceptable peak shape in HILIC mode. Indeed, samples in aqueous diluent (such as the AAVs) can generate breakthrough effects and peak distortion in HILIC mode as H_2_O has the highest eluotropic strength in this retention mechanism. To avoid these phenomena, a fast initial ramp (0.2 min) at high organic solvent composition (85% ACN) is generally used at the beginning of the gradient to compensate the effects of the aqueous sample diluent at the injection [29,30,31,32,33,34]. However, in the context of developing a multi-isocratic method using pre-mixed mobile phases, this approach is not applicable since both mobile phases will consist of 0.1% TFA in a mixture of H_2_O and organic solvent. For this reason, various injection volumes (0.2, 0.3, 0.4, and 0.5 μL) were screened in combination with pre-mixed mobile phases by using a 15 min linear gradient from 0 to 100%B. MPA consisted of 0.1% TFA in [ACN/IPA (80/20, *v*/*v*)]/H_2_O (75/25, *v*/*v*) and MPB was 0.1% TFA in [ACN/IPA (80/20, *v*/*v*)]/H_2_O (70/30, *v*/*v*). As shown in Figure S2, a breakthrough effect was observed when injecting 0.5 μL, and it was still observable (though at a lower intensity) when using 0.4 μL. Instead, the injection of 0.3 μL was found to be a perfect compromise to avoid the breakthrough effect while retaining the separation performance (that was corrupted when using 0.2 µL as injection volume).

**Figure 6:**
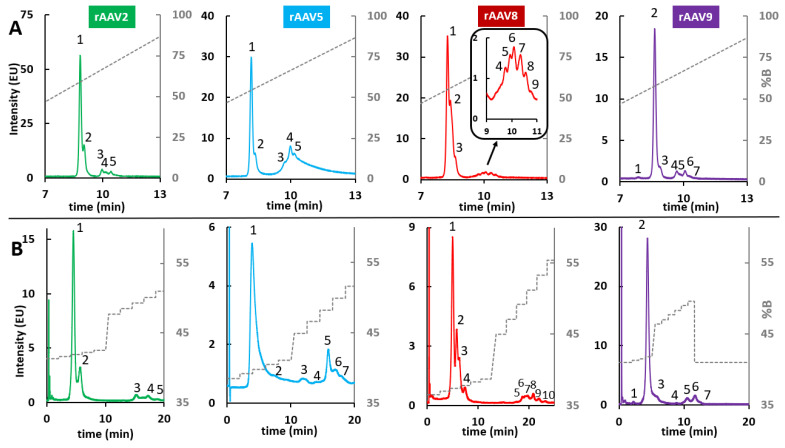
Effect of gradient type on HILIC analysis of rAAV VPs: comparison of (**A**) a linear gradient (15 min gradient 0–100%B) and a (**B**) multi-step gradient on separation efficiency on a Waters ACQUITY Glycoprotein BEH Amide column. MPA: [IPA/ACN (80/20, *v*/*v*)]/H_2_O (79/21, *v*/*v*) + 0.1% TFA; MPB: [ACN/IPA (80/20, *v*/*v*)]/H_2_O (67/33, *v*/*v*) + 0.1% TFA. Peak numbering has the aim of highlighting the different peaks that are separated when using a linear gradient versus a multi-step gradient.

Once the mobile phase composition, the column temperature, and the injection volume were defined, multi-isocratic elution methods were developed by following the workflow proposed by Murisier et al. [26]. In this case, some pre-mixed mobile phases were identified after gradient optimization. Specifically, MPA consisted of 0.1% TFA in [ACN/IPA (80/20, *v*/*v*)]/H_2_O (79/21, *v*/*v*) and MPB was 0.1% TFA in [ACN/IPA (80/20, *v*/*v*)]/H_2_O (67/33, *v*/*v*). These pre-mixed mobile phases were used for the application of both steps (ii) and (iii) of the workflow previously described and the corresponding chromatograms are shown in Figure 6. Detailed multi-isocratic gradients are reported in Table S3. Interestingly, the application of this methodology allowed up to 11 protein variants to be separated (in the case of VPs from AAV8) after stepwise optimization and, more generally, an improvement in selectivity of the set of peaks related to VP3 and those related to VP1 and VP2 was achieved in all cases. However, it should be mentioned that the optimization of multi-isocratic gradients is long, tedious, and non-generic (as the gradient needs to be adapted to each sample). Moreover, the final methods are rather complex and not very robust as the preparation of pre-mixed mobile phases can be a source of variability and the separation is extremely sensitive to small differences in mobile phase composition. Therefore, despite the improvement in VPs selectivity, it must be considered that this type of methodology is not easily compatible with the standards required in QC laboratories.

### 2.4. HIC

In addition to RPLC and HILIC, HIC is another chromatographic method that can be used to separate AAV capsid proteins. In fact, this chromatographic mode has been used in the past for the analysis and/or purification of various types of proteins. It consists of using a slightly hydrophobic stationary phase (often silica bonded with a low density of butyl ligand) and a purely aqueous mobile phase at physiological pH in the presence of a large amount of salts (usually up to 1–2 M ammonium sulfate). An inverse salt gradient is then applied to elute proteins based on their increasing hydrophobicity [35]. More information on the HIC retention mechanism [36] and applications [37] can be found elsewhere. Interestingly, HIC can be potentially coupled to MS, as recently demonstrated [38]. To the best of our knowledge, HIC has never been used for the separation of AAV capsid proteins.

**Figure 7:**
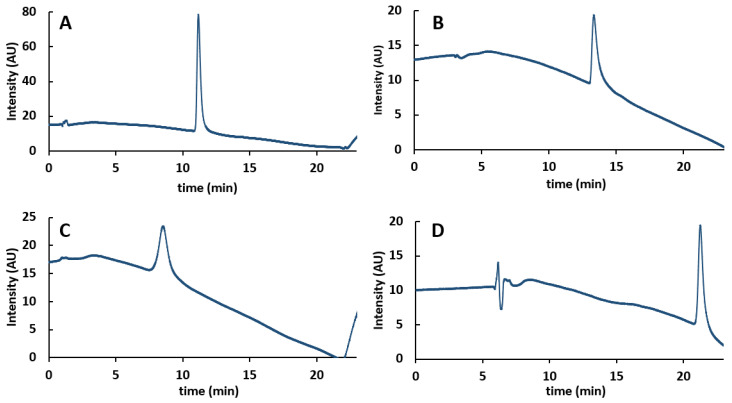
Evaluation of column performance: comparison of (**A**) BioPro HIC HT column, (**B**) TSK-Gel Butyl-NPR column, (**C**) TSK-Gel Ether-5PW column, and (**D**) mAbPac HIC-10 column for the analysis of AAV8 VPs with a 20 min gradient from 0 to 100%B. MPA: 100 mM phosphate buffer solution + 1.5 M (NH_4_)SO_4_, pH 6.8; MPB: 100 mM phosphate buffer solution, pH 6.8.

It is well known that the stationary phase can have a strong influence on protein separation in HIC [38]. Therefore, four different stationary phases (Table S1) were tested for the analysis of AAV8 VP using a generic mobile phase consisting of phosphate buffer at pH 6.8 and ammonium sulfate. The corresponding chromatograms are reported in Figure 7. As shown, the TSK-Gel Ether-5PW column was the least retentive column (ethyl bonding with limited hydrophobicity), while the mAbPac HIC-10 column was the most retentive one (alkylamide bonding with higher hydrophobicity). The other two columns, BioPro HIC HT and Tosoh TSK-Gel Butyl-NPR, have intermediate hydrophobicity (butyl bonding). These results are consistent with a previous study on mAbs showing a similar retention behavior [38]. In addition to retention, there were some significant differences in peak shape. A relatively symmetrical and narrow peak was observed on the BioPro HIC HT column, which is packed with the smallest particle size (2.3 µm), whereas the other three columns produced either tailed or broad peaks. The lowest efficiency was observed on the TSK-Gel Ether-5PW column, which is logical as this column is packed with 10 µm fully porous particles. Regardless of the column, it was not possible to separate VP1-3 species using the generic gradient conditions from 0 to 100%B. However, the most promising results were observed on the BioPro HIC HT column, which was selected for further investigation.

**Figure 8:**
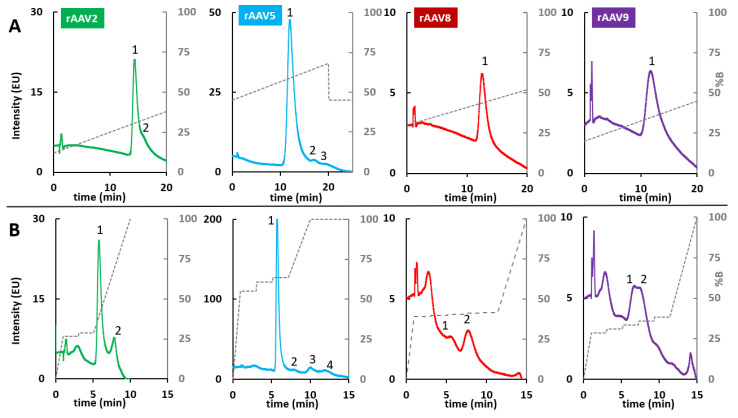
Effect of gradient type on HIC analysis of AAV VPs: comparison of (**A**) a linear gradient (15 min gradient from 12 to 38%B for AAV2, 45 to 68%B for AAV5, 30 to 52%B for AAV8 and 20 to 45%B for AAV9) and a (**B**) multi-step gradient on a BioPro HIC HT column. MPA: 100 mM phosphate buffer solution + 1.5 M (NH_4_)SO_4_, pH 6.8; MPB: 100 mM phosphate buffer solution, pH 6.8. Peak numbering has the aim of highlighting the different peaks that are separated when using a linear gradient versus a multi-step gradient.

Next, the initial and final gradient compositions were optimized for VPs obtained from the four AAV serotypes, while maintaining the gradient time at 20 min to maximize resolution. Due to differences in amino acid composition between the different VPs [39], the gradient conditions for VP elution must be adjusted on a case-by-case basis. It appears that VPs obtained from the AAV5 product require the largest proportion of %B (45–68%B) to elute the VP1-3 subunits, highlighting the higher hydrophobicity of the VPs in AAV5 product. On the other hand, the %B in the HIC mobile phase was lower for the VPs obtained from the other three serotypes, with 30–52%B for AAV8, 20–45%B for AAV9, and 12–38%B for AAV2. The corresponding chromatograms obtained under the conditions described above are shown in Figure 8A.

As shown, despite the optimization of the gradient conditions, only a single, relatively broad peak was observed for the AAV8 and AAV9 products, indicating that the differences in hydrophobicity between VP1-3 were not sufficient to allow a separation under HIC conditions. On the other hand, a slight separation between two species was observed for AAV2, and three peaks were distinctly separated for the AAV5 serotype.

To further improve the separation between VP species, multi-step gradients were developed for each AAV serotype, similarly to the RPLC and HILIC modes. The corresponding chromatograms are shown in Figure 8B. Detailed multi-isocratic gradients are reported in Table S4. For AAV2 and AAV5, the retention times of the observed peaks were converted into the elution composition and some isocratic steps were added to these particular compositions, as described elsewhere [40]. This strategy significantly improved the separation of the two species observed for AAV2. The result was even more remarkable for AAV5, with the appearance of an additional peak compared to the linear gradient. However, the optimization was time-consuming and requires some adjustment to find out the optimal separation.

The optimization procedure was different for AAV8 and AAV9. For these two serotypes, only a single peak was observed with the linear gradient. Therefore, the retention times at the start and end of the peaks were converted into elution compositions. Inside this elution window (from the start to the end of the peak), five isocratic steps were included to maximize the resolving power, as described elsewhere [26]. In the end, two peaks were detected for AAV8, but with a very low sensitivity. The single peak observed for AAV9 with the linear gradient was split into two species partially separated when using the multi-step gradient, but again sensitivity was strongly reduced.

Finally, the resolving power of HIC was far below the performance of RPLC and HILIC. In addition, the hyphenation of HIC with MS is more complex to implement. For these reasons, it appears that HIC is not a viable solution for the analytical characterization of VP species, despite a full optimization of the HIC conditions.

## 3. Materials and Methods

### 3.1. Chemical and Reagents

Type 1 water was provided by a Milli-Q purification system from Millipore (Bedford, MA, USA). 3-(2-furoyl) quinoline-2-carboxaldehyde (ATTO-TAG™FQ derivatization reagent), which was abbreviated as FQ-Dye in the text, Potassium cyanide (KCN), Acetonitrile (ACN), isopropanol (IPA), ethanol (EtOH), methanol (MeOH), formic acid (FA), andtrifluoroacetic acid (TFA) were purchased from Fisher Scientific (Reinach, Switzerland). NEM (N-Ethylmaleimid), Dimethylsulfoxide (DMSO), Acetic acid (glacial, ReagentPlus^®^, >99%), potassium phosphate monobasic (BioUltra for molecular biology, anhydrous, >99.5%), and potassium phosphate dibasic (BioUltra for molecular biology, anhydrous, >99.0%) were obtained from Sigma-Aldrich (Buchs, Switzerland). Ammonium sulfate was purchased from AppliChem GmbH (Darmstadt, Germany). SDS-MW Gel Buffer was provided by AB Sciex (Framingham, MA, USA). SDS-Solution (10%) was obtained from BioRad (Hercules, CA, USA) and 0,1 mol/L Sodium hydroxide (NaOH) and 0.1 mol/L hydrochloric acid (HCl) purchased from Titripur^®^ Supelco (Bellefonte, PA, USA). AAVr samples were provided by Roche (Penzberg, Germany). The concentration of rAAV samples is expressed as the number of viral particles per milliliter (vp/mL). In the present work, rAAV samples were obtained at different concentrations (2 × 10^13^ vp/mL for serotypes 2, 8, and 9, and 4 × 10^13^ vp/mL for serotype 5) from either Virovek (Hayward, CA, USA) or from Sirion Biotech (Graefelfing, Germany) and stored at −80 °C.

### 3.2. Sample Preparation

Prior to CE-SDS analysis, 15 µL of AAV8 sample solution was mixed with 1.8 µL of 4% SDS in 150 mM NEM solution in a 0.65 mL micro-centrifuge tube and incubated at 70 °C for 5 min. The sample solution was then mixed with 2.25 µL of 2.5 mM FQ dye working solution and 1.5 µL of 30 mM KCN solution and incubated at 70 °C for 10 min. Then, 42 µL of 1% SDS was added to quench the labelling reaction. The sample solution was incubated at 70 °C for 5 min. Finally, 30 µL of DI water was added to the mixture. No buffer exchange or desalting procedure was performed. The diluted mixture was finally transferred to the sample vial for analysis. The same procedure was applied for all other serotypes and the samples were used undiluted, even if the concentrations of the samples were different.

Prior to LC analysis, rAAV samples were diluted to 1 × 10^13^ vp/mL and treated with acetic acid at 10% (*v*/*v*) concentration for 15 min. All samples were stored at 4 °C after the sample preparation and analyzed within 72 h.

### 3.3. Instrumentation and Experimental Conditions

#### 3.3.1. CE-SDS Analysis

CE-SDS analyses of VPs obtained from different rAAV serotypes were performed on a PA800 Plus Pharmaceutical Analysis CE system (SCIEX, Framingham, MA, USA) equipped with a laser-induced fluorescence (LIF) detector (488 nm excitation with 600 nm emission filter). CE-SDS separations were performed at 20 cm separation distance (bare fused silica capillary 50 µm inner diameter, 30 cm total length). All separations were performed at 25 °C. Samples were stored at 10 °C. A reverse polarity (cathode on the injection side) was employed, and voltage of 15 kV was applied. The samples were injected at 5.0 kV for 60 s (with a pre-injection of water at 20.0 psi for 0.4 min). The separating capillary was rinsed with 0.1 M NaOH for 10 min, 0.1 M HCl for 5 min, MilliQ-Water for 2 min, and finally SDS-MW separating matrix for 10 min. Data acquisition and analysis were performed using 32 Karat^TM^ Software 10. These reference conditions were adapted from [23].

#### 3.3.2. LC Analysis

LC analyses of VPs obtained from different rAAV serotypes were performed on an ACQUITY UPLC H-Class system (Waters, Milford, MA, USA), equipped with an auto-sampler, and a binary solvent delivery pump. Data were acquired with a fluorescence detector (FD) using an excitation wavelength of 280 nm and emission at 345 nm. Samples were stored at 6 °C. Data acquisition and instrument control were performed using Empower Pro 3 software (Waters).

##### RPLC Conditions

Preliminary method optimization to select appropriate stationary phase and mobile phase additives was performed by using viral proteins (VPs) obtained from rAAV8. Mobile phases consisted of either 0.1% TFA, 0.1% FA, or 1% FA in H_2_O (MPA) and ACN (MPB). A generic linear gradient from 20% to 60% B in 10 min (4.0%B/min) was applied for the stationary phase and additive screening. All the columns were operated under the same conditions, but due to its pressure limit (~200 bar), the ProSwift column was used at a lower flow rate. Investigated RPLC columns are reported in Table S1. Once the stationary phase and mobile phase additive were selected, several organic modifiers (i.e., ACN, IPA, or EtOH) were tested using optimized gradient conditions (0.15%B/min). Finally, various linear and multi-step gradients were optimized for the different serotypes as described in the results and discussion section. Optimized RPLC multi-step gradients are given in Table S2.

##### HILIC Conditions

As starting conditions, 0.5 μL of rAAV8 was injected on an ACQUITY Glycoprotein BEH Amide column (1.7 μm, 2.1 × 50 mm) at 40 °C with a flow rate of 0.4 mL/min, and mobile phases composed of 0.1% TFA in H_2_O (MPA) and 0.1%TFA in ACN (MPB). Then, various column temperatures (70, 60, 50, 40, and 30 °C) were tested using a 12 min gradient from 70 to 60%B. Once the temperature was selected, various organic modifiers were tested, including ACN, ACN/IPA (80/20, *v*/*v*), or ACN/MeOH (80/20, *v*/*v*). Then, various sample volumes (0.2, 0.3, 0.4, and 0.5 μL) were injected using a 15 min linear gradient. Finally, various linear and multi-step gradients were optimized for the different serotypes as described in the results and discussion section. Optimized HILIC multi-step gradients are given in Table S3.

##### HIC Conditions

Some generic HIC conditions, usually applied for mAbs, were initially tested [41]. HIC mobile phases were prepared by using two independent solutions of 100 mM potassium phosphate monobasic and 100 mM potassium phosphate dibasic. MPA consisted of H_2_O containing 100 mM potassium phosphate buffer (potassium phosphate monobasic/potassium phosphate dibasic solutions, 20/80, *v*/*v*) and 1.5 M of ammonium sulfate, pH 6.8. MPB consisted of H_2_O containing 100 mM of potassium phosphate buffer (potassium phosphate monobasic/potassium phosphate dibasic solutions, 50/50, *v*/*v*), pH 6.8. Both mobile phases were filtered through PES 0.22 μm membrane filters before use.

First, several HIC columns were investigated, as listed in Table S1. Columns were operated at 45 °C with a flow rate of 0.2 mL/min (injection volume of 1 μL). Preliminary method optimization to select a suitable column was performed by using viral proteins (VPs) obtained from rAAV8. A generic linear gradient from 0 to 100%B in 20 min was sued for the column screening. Once the optimal column was selected, several temperatures (25, 45, and 60 °C) were tested. Finally, various linear and multi-step gradients were optimized for the different serotypes as described in the results and discussion section. Optimized RPLC multi-step gradients are given in Table S4.

## 4. Conclusions

Different electrophoretic and chromatographic methods were compared for the characterization of viral proteins of several AAV serotypes. CE-SDS was found to be particularly suitable for the size-based analysis of VP1-3 proteins but requires dedicated capillary electrophoresis instrumentation, including LIF detection. In addition, the minor impurities (oxidation, phosphorylation) present in the product remain difficult to separate and identify in CE-SDS due to the inherent incompatibility of CE-SDS with MS.

RPLC can be used instead of CE-SDS for the separation of VPs. In the present work, the RPLC method was first optimized in terms of stationary phase chemistry and mobile phase composition (wide pore ACQUITY BEH C4 column in combination with 0.1% TFA as mobile phase additive and ACN as organic modifier). Then, the optimization of the gradient profile allowed a better resolution of VP1 and VP2 for the rAAV8 product, where partially co-eluted peaks were baseline separated during the isocratic steps.

HILIC has also been evaluated as a chromatographic method for AAVs. To the best of our knowledge, HILIC has only been reported in one single publication. Here, the HILIC method was optimized in terms of column temperature, mobile phase composition, injection volume, and gradient profile. The best conditions were different from those reported in the literature. HILIC was found to be very complementary to RPLC, and an improvement in selectivity was achieved using multi-isocratic gradients, allowing the separation of up to 11 protein variants in the case of AAV8. However, the optimization of multi-isocratic gradients is long, tedious, and non-generic. Despite this improvement in the selectivity of VPs, this type of methodology is not easily compatible with the standards required in QC laboratories.

Finally, it was found that despite optimization of stationary phase chemistry and use of multi-isocratic gradient conditions, HIC offers disappointing performance for the separation of AAV capsid proteins.

## Data Availability

Not applicable.

## Supplementary Materials

The following supporting information can be downloaded at: https://www.mdpi.com/article/10.3390/ijms24108503/s1.
