# Supplementary material for: Boosting the Separation of Adeno-Associated Virus Capsid Proteins by Liquid Chromatography and Capillary Electrophoresis Approaches
Source: Int J Mol Sci. 2023 May 9;24(10):8503. doi: 10.3390/ijms24108503 (PMC10218170; doi:10.3390/ijms24108503)
Supplement: Supplementary file 1 [file ijms-24-08503-s001.zip › ijms-2380228-supplementary.pdf]

Supplementary Information

Boosting the Separation of Adeno-Associated Virus Capsid Proteins by Liquid Chromatography and Capillary Electrophoresis Approaches

Megane K. Aebischer [1,2,†], Thomas Bouvarel [1,2,†], Emmalyn Barrozo [1,2], Dominik Kochardt [3], Carsten Elger [3], Markus Haindl [3], Raphael Ruppert [3], Davy Guillarme [1,2] and Valentina D'Atri [1,2,*]

[1] School of Pharmaceutical Sciences, University of Geneva, CMU-Rue Michel Servet 1, 1211 Geneva, Switzerland
[2] Institute of Pharmaceutical Sciences of Western Switzerland, University of Geneva, CMU-Rue Michel Servet 1, 1211 Geneva, Switzerland
[3] Roche Diagnostics GmbH, Nonnenwald 2, 82377 Penzberg, Germany
* Correspondence: valentina.datri@unige.ch
† These authors contributed equally to this work.

TABLE OF CONTENTS

**Table S1**	List of investigated columns and their properties	S1
**Table S2**	Optimized RPLC multi-step gradients applied for the analyses of VPs obtained from different rAAV serotypes	S2
**Table S3**	Optimized HILIC multi-step gradients applied for the analyses of VPs obtained from different rAAV serotypes	S3
**Table S4**	Optimized HIC multi-step gradients applied for the analyses of VPs obtained from different rAAV serotypes	S4
**Figure S1**	Effect of solvent selection on RPLC analysis of rAAV8 VPs	S5
**Figure S2**	Effect of injection volume on HILIC analysis of rAAV8 VPs	S6

Table S1. List of investigated columns and their properties.

LC Mode	Name	Provider	Column chemistry	Column dimensions (mm)	Particle (µm) and pore (Å) size
RPLC	ACQUITY BEH C4	Waters	C4	2.1 x 150	1.7 µm, 300Å
	BioResolve RP mAb	Waters	Polyphenyl	2.1 x 50	2.7 µm, 450 Å
	YMC-Triart Bio C4	YMC	Bioinert C4	2.1 x 50	1.7 µm, 300Å
	ProSwift RP-4H	Thermo	Polymeric	1.0 x 50	Monolith
HILIC	ACQUITY Glycoprotein BEH Amide	Waters	Amide	2.1 x 150	1.7 µm, 300Å
HIC	BioPro HIC HT	YMC	Butyl	4.6 x 33	2.3 µm
	MAbPac HIC-10	Thermo	Alkylamide	4.6 x 100	5.0 µm, 1000Å
	TSK-Gel Ether-5PW	Tosoh	Ethyl	2.0 x 75	10 µm, 1000Å
	TSK-Gel Butyl-NPR	Tosoh	Butyl	4.6 x 100	2.5 µm

Table S2. Optimized RPLC multi-step gradients applied for the analyses of VPs obtained from different rAAV serotypes, namely rAAV2, rAAV5, rAAV8, and rAAV9. MPA: H_2O/ACN (66/34 for AAV8 and 65/35 for AAV2, AAV5, and AAV9) + 0.1% TFA; MPB: H_2O/ACN (64/36 for AAV8 and 63/37 for AAV2, AAV5, and AAV9) + 0.1% TFA.

**VPs from rAAV2**		**VPs from rAAV5**		**VPs from rAAV8**		**VPs from rAAV9**	
**Time (min)**	%B	Time (min)	%B	Time (min)	%B	Time (min)	%B
**0**	0	0	0	0	0	0	0
**1.0**	23.9	1.0	39.3	1.0	37.7	1.0	40.4
**3.0**	23.9	3.0	39.3	3.0	37.7	3.0	40.4
**3.1**	29.7	3.1	44.5	3.1	41.6	3.1	44.6
**5.1**	29.7	5.1	44.5	5.1	41.6	5.1	44.6
**5.2**	33.1	5.2	46.1	5.2	51.4	5.2	45.4
**7.2**	33.1	7.2	46.1	7.2	51.4	7.2	45.4
**10.0**	100	7.3	49.2	7.3	54.0	7.3	53.5
		9.3	49.2	9.3	54.0	9.3	53.5
		10.0	100	9.4	58.9	9.4	57.3
				11.4	58.9	11.4	57.3
				11.5	61.3	11.5	62.9
				13.5	61.3	13.5	62.9
				15.0	100	15.0	100

Table S3. Optimized HILIC multi-step gradients applied for the analyses of VPs obtained from different rAAV serotypes, namely rAAV2, rAAV5, rAAV8, and rAAV9. MPA: [ACN/IPA (80/20, v/v)] / H_2O (79/21, v/v) + 0.1% TFA; MPB: [ACN/IPA (80/20, v/v)] / H_2O (67/33, v/v) + 0.1% TFA.

**VPs from rAAV2**		**VPs from rAAV5**		**VPs from rAAV8**		**VPs from rAAV9**	
**Time (min)**	%B	Time (min)	%B	Time (min)	%B	Time (min)	%B
**0**	41.3	0	38.5	0	36.5	0	41.0
**2.00**	41.3	2.00	38.5	2.50	36.5	2.00	41.0
**2.01**	41.6	2.01	39.2	2.51	37.0	2.01	41.3
**4.01**	41.6	4.01	39.2	4.51	37.0	3.01	41.3
**4.02**	41.9	4.02	39.8	4.52	37.4	3.02	41.5
**6.02**	41.9	6.02	39.8	6.52	37.4	4.02	41.5
**6.03**	42.2	6.03	40.5	6.53	37.8	4.03	41.8
**8.03**	42.2	8.03	40.5	8.53	37.8	5.03	41.8
**8.04**	42.5	8.04	41.1	8.54	38.3	5.53	46.4
**10.04**	42.5	10.04	41.1	10.54	38.3	6.53	46.4
**10.54**	47.7	10.54	44.9	10.55	38.7	6.54	47.0
**12.54**	47.7	12.54	44.9	12.55	38.7	7.54	47.0
**12.55**	48.5	12.55	46.6	13.55	45.0	7.55	47.7
**14.55**	48.5	14.55	46.6	15.55	45.0	8.55	47.7
**14.56**	49.3	14.56	48.2	15.56	47.1	8.56	48.3
**16.56**	49.3	16.56	48.2	17.56	47.1	9.56	48.3
**16.57**	50.1	16.57	49.9	17.57	49.2	9.57	49.0
**18.57**	50.1	18.57	49.9	19.57	49.2	10.57	49.0
**18.58**	51.0	18.58	51.6	19.58	51.2	10.58	49.6
**20.58**	51.0	20.58	51.6	21.58	51.2	11.58	49.6
**20.59**	51.8	20.59	53.2	21.59	53.3	11.6	41.0
**22.59**	51.8	22.59	53.2	23.59	53.3	20.0	41.0
**22.6**	41.3	22.6	38.5	23.6	55.4		
**30.0**	41.3	30.0	38.5	26.1	55.4		
				26.2	36.5		
				40.0	36.5		

Table S4. Optimized HIC multi-step gradients applied for the analyses of VPs obtained from different rAAV serotypes, namely rAAV2, rAAV5, rAAV8, and rAAV9. MPA: 100 mM phosphate buffer solution + 1.5 M $(NH_4)SO_4$, pH 6.8; MPB: 100 mM phosphate buffer solution, pH 6.8.

**VPs from rAAV2**		**VPs from rAAV5**		**VPs from rAAV8**		**VPs from rAAV9**	
**Time (min)**	%B	Time (min)	%B	Time (min)	%B	Time (min)	%B
**0**	0	0	0	0	0	0	0
**1.0**	26.5	1.0	55.1	1.0	39.0	1.0	28.5
**3.0**	26.5	3.0	55.1	3.0	39.0	3.0	28.5
**3.1**	28.6	3.1	60.8	3.1	39.7	3.1	31.0
**5.1**	28.6	5.1	60.8	5.1	39.7	5.1	31.0
**10.0**	100	5.2	63.6	5.2	40.3	5.2	33.5
		7.2	63.6	7.2	40.3	7.2	33.5
		10.0	100	7.3	41.0	7.3	36.0
				9.3	41.0	9.3	36.0
				9.4	41.4	9.4	38.5
				11.4	41.4	11.4	38.5
				15.0	100	15.0	100

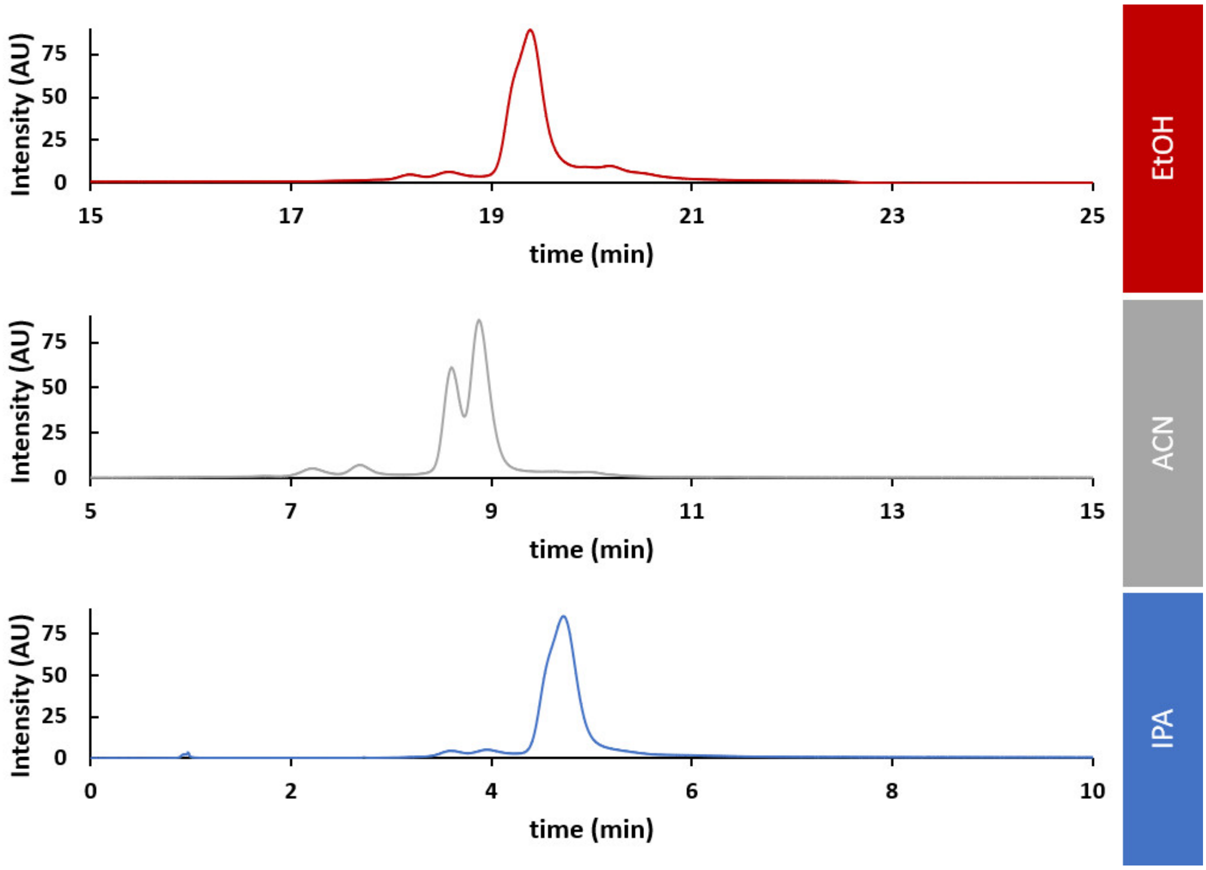

Figure S1. Effect of solvent selection on RPLC analysis of rAAV8 VPs. Comparison of IPA, ACN, and MeOH mobile phases on an Acquity UPLC Protein BEH C4 column (flow rate 0.4 mL/min, 2 µL injected, 70°C) with a 20 min gradient from 27 to 30 %B for IPA, 36 to 39 %B for ACN, and 38 to 41 %B for EtOH). MPA: H_2O + 0.1% TFA; MPB: EtOH (red), ACN (grey), or IPA (blue) + 0.1% TFA.

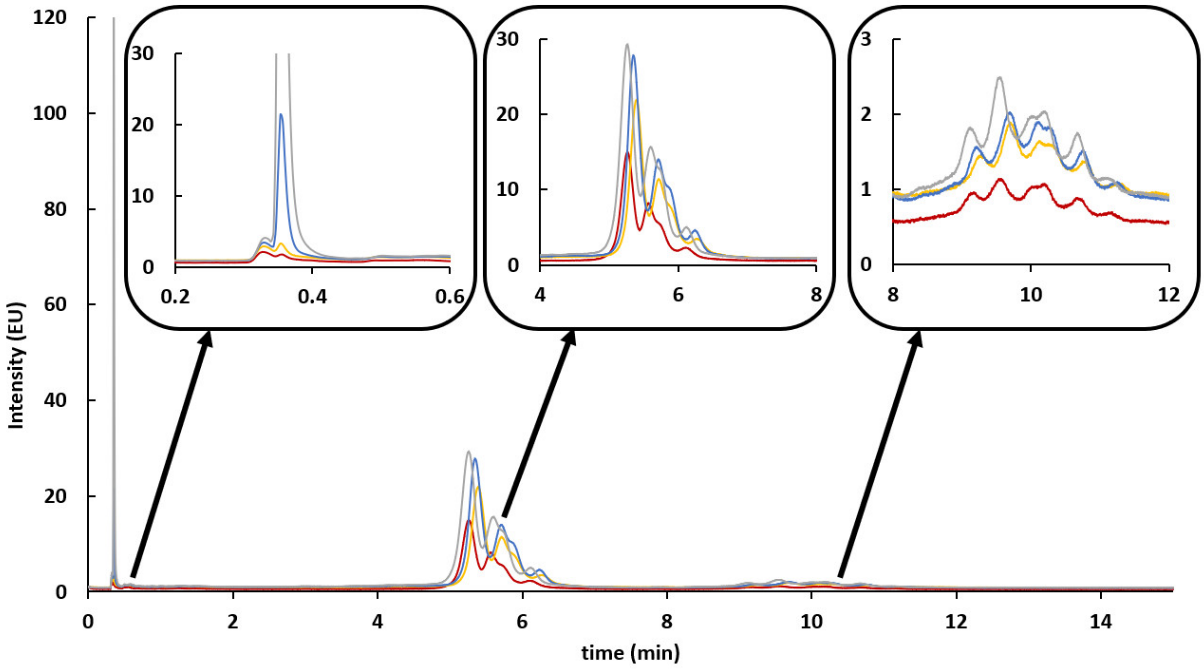

Figure S2. Effect of injection volume on HILIC analysis of rAAV8 VPs: comparison of 0.2 µL (red), 0.3 µL (yellow), 0.4 µL (blue), and 0.5 µL (grey) on Waters ACQUITY Glycoprotein BEH Amide column with a 15 min gradient from 0 to 100 %B. MPA: [ACN/IPA (80/20, v/v)] / H_2O (75/25, v:v) + 0.1%TFA; MPB: [ACN/IPA (80/20, v/v)] / H_2O (70/30, v/v) + 0.1% TFA.
